# Association between survival and levetiracetam use in glioblastoma patients treated with temozolomide chemoradiotherapy

**DOI:** 10.1038/s41598-020-67697-w

**Published:** 2020-07-01

**Authors:** Tae Hoon Roh, Ju Hyung Moon, Hun Ho Park, Eui Hyun Kim, Chang-Ki Hong, Se Hoon Kim, Seok-Gu Kang, Jong Hee Chang

**Affiliations:** 1 Department of Neurosurgery, Ajou University Hospital, Ajou University School of Medicine, Suwon, Republic of Korea; 2 Department of Neurosurgery, Severance Hospital, Yonsei University College of Medicine, 50 Yonsei-ro, Seodaemun-gu, Seoul, 03722 Republic of Korea; 3 Department of Neurosurgery, Gangnam Severance Hospital, Yonsei University College of Medicine, Seoul, Republic of Korea; 4 Department of Pathology, Severance Hospital, Yonsei University College of Medicine, Seoul, Republic of Korea

**Keywords:** CNS cancer, Disease-free survival

## Abstract

This study was conducted to assess whether levetiracetam (LEV) affects the survival of patients with glioblastoma (GBM) treated with concurrent temozolomide (TMZ) chemotherapy. To this end, from 2004 to 2016, 322 patients with surgically resected and pathologically confirmed isocitrate dehydrogenase (*IDH*)-wildtype GBM who received TMZ-based chemoradiotherapy were analysed. The patients were divided into two groups based on whether LEV was used as an anticonvulsant both at the time of surgery and the first visit thereafter. The median overall survival (OS) and progression-free survival (PFS) were compared between the groups. The OS was 21.1 and 17.5 months in the LEV (+) and LEV (−) groups, respectively (P = 0.003); the corresponding PFS was 12.3 and 11.2 months (P = 0.017). The other prognostic factors included age, extent of resection, O^6^-methylguanine-DNA methyltransferase (*MGMT*) promoter methylation status, and Karnofsky Performance Status (KPS) score. The multivariate analysis showed age (hazard ratio [HR], 1.02; P < 0.001), postoperative KPS score (HR 0.99; P = 0.002), complete tumour resection (HR 0.52; P < 0.001), *MGMT* promoter methylation (HR 0.75; P < 0.001), and LEV use (HR 0.72; P = 0.011) were significantly associated with OS. In conclusion, LEV use was associated with prolonged survival in patients with GBM treated with concurrent TMZ chemoradiotherapy.

## Introduction

Glioblastoma (GBM) is one of the most malignant brain tumours. To improve the survival of patients with GBM, surgery should be performed to remove as much of the tumour as possible, followed by chemotherapy and radiotherapy^1, 2^. Despite the standard therapy, the median overall survival (OS) of patients with GBM is only 14.6–20.2 months^2–4^. Temozolomide (TMZ), the chemotherapy agent for GBM, is an alkylating agent that causes the methylation of DNA, resulting in anticancer effects^5^, and GBMs with methylated O^6^-methylguanine DNA methyltransferase (*MGMT*) promoters are more sensitive to TMZ^5, 6^.

Approximately 30–50% of patients with brain tumours present with seizures, and 29–49% of patients with GBM experience seizures^7^. However, the need for prophylactic antiepileptic drug (AED) therapy in asymptomatic patients remains controversial. Several neurosurgeons use prophylactic AEDs for various durations after brain tumour surgery^8–11^. Because AEDs are often combined with chemotherapy, it has been a topic of interest to identify AEDs that are most beneficial when used concomitantly with TMZ.

Seizures reduce the quality of life of patients with glioma. Our treatment policy for patients with GBM was to use prophylactic AEDs and maintain it for as long as possible unless adverse drug reactions occur. In our institution, valproic acid (VPA) has been the first-choice AED since 2004. Since 2010, levetiracetam (LEV) gradually replaced VPA. LEV belongs to a newer group of AEDs that do not induce or inhibit cytochrome P450^12^. Currently, it is widely used as it causes fewer adverse events and drug interactions^8, 9, 13–19^.

A recent study showed that LEV inhibited *MGMT* and sensitised GBM cells to TMZ^20^. Another study showed that patients who received LEV in combination with TMZ had longer OS and progression-free survival (PFS) than those who did not receive LEV^21^. Therefore, in this study, we investigated whether the use of LEV afforded any survival benefit.

## Methods

### Patient recruitment

From 2004 to 2016, 429 consecutive patients with surgically resected and pathologically confirmed GBM treated with TMZ-based chemoradiotherapy in Yonsei University Severance Hospital in Seoul, Korea, were retrospectively reviewed. Patients younger than 18 years of age and those who received only radiation or only chemotherapy were excluded from this study. All patients were tested for the *IDH1* mutation. As the *IDH2* mutations are rare in adults with GBM, we did not test for these. Eighteen of 340 patients with GBMs were found to have the *IDH1* mutation and were excluded from the analysis. Finally, 322 patients were included in the study. This retrospective human study was approved by the institutional review board of Yonsei University Severance Hospital (Approval no. 4-2019-1069), which waived the requirement of obtaining informed consent. All methods were performed in accordance with the relevant guidelines and regulations.

During the study period, ten neurosurgeons performed glioma surgery at our institution. The institution’s policy was to administer AEDs (selected according to the preference of the surgeon) upon initial discovery of GBM tumour-induced seizures. After tumour resection, AEDs were administered as prophylaxis regardless of seizure history. AEDs were administered as monotherapy unless the patient presented a new seizure event. As a starting dose, 1,000 mg/day of VPA or LEV was administered. If a seizure event occurred thereafter, 500 mg/day of VPA or LEV was added. If additional seizure events occurred, other AEDs were added. In patients with neuralgia, 900 mg/day of gabapentin or 150 mg/day of pregabalin was added and escalated by symptoms. That therapy was maintained unchanged unless serious adverse events occurred. Information on duration of maintenance on the initial prophylactic AED therapy was collected.

The patients were divided into two groups based on whether LEV was used as the anticonvulsant during chemoradiotherapy and adjuvant chemotherapy. If LEV was included and administered at both baseline and the first visit after the concomitant phase of chemoradiotherapy, the patients were classified into the ‘LEV (+)’ group. Patients who were not taking LEV on both occasions were classified into the ‘LEV (−)’ group.

### Surgery and adjuvant treatment

Postoperative magnetic resonance imaging (MRI) was performed for all patients within 72 h after the surgery. The extent of resection was determined based on the MRI findings. Complete resection was defined as the absence of an enhancing lesion on T1-weighted contrast-enhanced images. If there were existing residual tumours in the operation field, the patient was considered to have undergone an incomplete resection even if postoperative MRI showed no residual tumour.

Concomitant chemoradiotherapy (CCRT) with TMZ followed by adjuvant TMZ therapy according to Stupp’s regimen was started for all patients^3^. The patients received radiation therapy at a total dose of 60 Gy, with daily fractions of 2 Gy. The follow-up MRI was performed after CCRT, and then after the third and sixth cycles of TMZ. Thereafter, MRI was performed every 3–6 months to assess the disease status. The Response Assessment in Neuro-Oncology criteria were used to determine disease progression^22^. During chemoradiotherapy and adjuvant chemotherapy, treatment-related adverse events were graded according to the National Cancer Institute Common Terminology Criteria for Adverse Events ver. 4.0.3.

### Molecular analysis

The *MGMT* promoter methylation status was tested using a methylation specific-polymerase chain reaction (PCR) method as described previously^6^. The *IDH1* mutation was tested using immunohistochemistry with anti-human IDH1 R132H mouse monoclonal antibody (clone H09L, Dianova, 1:80 dilution), peptide nucleic acid-mediated PCR clamping, or a direct sequencing method.

### Statistical analysis

Categorical variables were compared using the chi-square test or Fisher’s exact test. Continuous variables were compared using Student’s *t* test, assuming equal variance, and P values were calculated using a two-tailed test. Mann–Whitney test was used for non-parametric statistics. Survival was analysed using the Kaplan–Meier method and compared using the log-rank test. OS was defined as the time from first surgery until death. PFS was defined as the time from the first surgery until disease progression (as confirmed by radiologic study) or death. The reverse Kaplan–Meier estimator was used for measuring the duration of follow-up and duration of AED administration. The Cox-proportional hazards model with a backward stepwise method was used for multivariate analysis. The results with a P value of < 0.05 were considered statistically significant.

## Results

Patient characteristics are summarised in Table 1. The median age of the patients was 58 (range 19–79) years. The patients included 141 women and 181 men. Seizures were the presenting symptom in 33 patients (10%). The median preoperative and postoperative Karnofsky Performance Status (KPS) scores were 80 (range 30–100) and 70 (range 10–100), respectively. One hundred and seventy-one patients (53%) had undergone complete resection. LEV was the most frequently used AED (169 patients, 53%) and used at 500 mg twice a day in most cases. The second most frequently used AED was VPA (132 patients, 42%) at a dose of 500 mg twice a day. One patient in the LEV (+) group received both LEV and VPA. The *MGMT* promoter methylation status was available for 296 (92%) patients. Among them, 87 (29%) showed methylation of the *MGMT* promoter. The median follow-up period was 60.8 (95% confidence interval [CI] 52.2–69.4) months and the median duration of AED administration was 37.3 (95% CI 22.8–51.8) months, as determined using the reverse Kaplan–Meier method. For the entire cohort (322 patients), the median OS and PFS were 19.4 (95% CI 16.9–21.9) and 12.0 (95% CI 10.8–13.1) months, respectively. Two hundred and fifty-five (79%) patients died by the time of analysis. Tumour progression was observed in 282 (88%) patients by the time of analysis.

**Table 1:** Patient demographics (N = 322).

Parameter	No. of patients (%)
Median age at diagnosis, years (range)	58 (19–79)
**Sex**	
Male	181 (56)
Female	141 (44)
Seizure as a presenting symptom	33 (10)
**Median KPS score (range)**	
Preoperative	80 (30–100)
Postoperative	70 (10–100)
**Extent of resection**	
Complete	171 (53)
Incomplete	181 (47)
**AED during CCRT and adjuvant chemotherapy**	
LEV included	169 (53)
LEV only	157 (49)
LEV plus others	12 (4)
LEV not included	153 (48)
Valproic acid only	111 (35)
Valproic acid plus others	21 (7)
Others	20 (6)
No AED	1 (0)
**MGMT promoter methylation**	
Methylated	87 (29)
Unmethylated	209 (71)
Not available	26
Median follow-up duration, months (95% CI)	60.8 (52.2–69.4)
Median AED maintenance duration, months (95% CI)	37.3 (22.8–51.8)
Overall survival, months (95% CI)	19.4 (16.9–21.9)
Dead	255 (79)
Progression-free survival, months (95% CI)	12.0 (10.8–13.1)
Tumour progression	282 (88)

*KPS *Karnofsky Performance Status, *AED* antiepileptic drug, *CCRT* concurrent chemoradiotherapy, *LEV* levetiracetam, *MGMT* O^6^-methylguanine-DNA methyltransferase, *CI* confidence interval.

Table 2 shows the characteristics of each group of patients. One hundred and sixty-nine (52%) patients received LEV during CCRT. The age and sex of the patients, preoperative history of seizure, and *MGMT* promoter methylation status were not significantly different between the two groups. The LEV (+) group showed higher preoperative and postoperative KPS scores than the LEV (−) group. Furthermore, the LEV (+) group was more likely to undergo complete resection than the control group (63% vs 42%, P < 0.001). The median duration of AED maintenance was 31.3 months (95% CI 19.9–42.6 months) in the LEV (+) group. The median duration of AED maintenance in the LEV (−) group was not obtained because most patients were administered AEDs until death.

**Table 2:** Patient demographics according to the use of levetiracetam.

Parameters	No. of patients (%)		P value
	LEV (+)	LEV (−)	
	n = 169	n = 153	
Age: mean ± SD	55 ± 12	57 ± 13	0.184
Men	97 (57)	84 (55)	0.652
Seizure as a presenting symptom	22 (13)	11 (7)	0.085
Preoperative KPS: mean ± SD	77 ± 15	72 ± 14	0.001
Postoperative KPS: mean ± SD	72 ± 18	68 ± 17	0.044
Complete resection	106 (63)	65 (42)	< 0.001
Methylated MGMT promoter	47/164 (29)	40/132 (30)	0.758

*LEV *levetiracetam, *SD* standard deviation, *KPS* Karnofsky Performance Status, *MGMT* O^6^-methylguanine-DNA methyltransferase.

**Figure 1:**
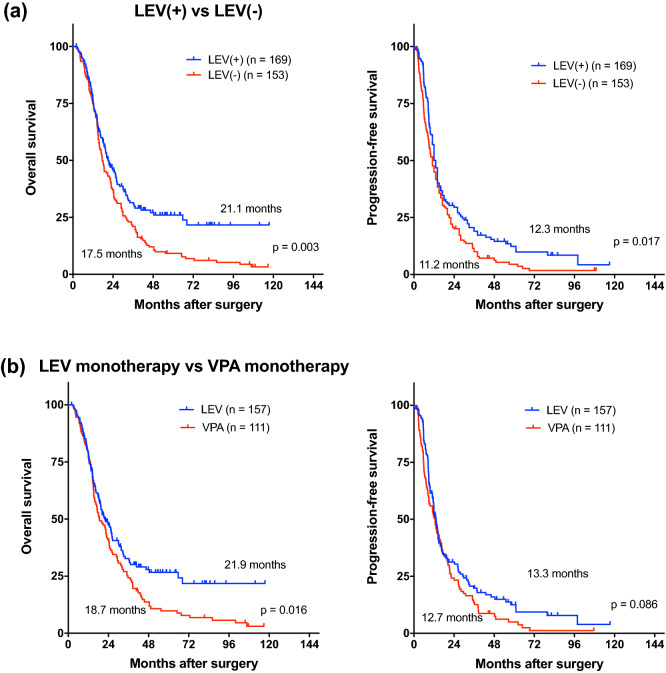
(**a**) Kaplan–Meier survival curves showing the overall survival and progression-free survival of patients. The overall survival was 21.1 months in the LEV (+) group and 17.5 months in the LEV (−) group (P = 0.003). The progression-free survival was 12.3 months in the LEV (+) group versus 11.2 months in the LEV (−) group (P = 0.017). (**b**) Comparison of the LEV monotherapy group and VPA monotherapy group. The overall survival was 21.9 months in LEV monotherapy group and 18.7 months in VPA monotherapy group (P = 0.016). Progression-free survival was 13.3 months in the LEV group and 12.7 months in the VPA group (P = 0.082).

The median OS in the LEV (+) and LEV (−) groups was 21.1 (95% CI 7.1–25.1) and 17.5 (95% CI 14.5–20.5) months, respectively (P = 0.003). The corresponding median PFS was 12.3 (95% CI 10.6–13.9) and 11.2 (95% CI 8.9–13.6) months (P = 0.017) (Fig. 1a).

Additionally, we compared the LEV monotherapy group (157 patients) with the VPA monotherapy group (111 patients). The median OS was 21.9 months (95% CI 17.3–26.5) in the former and 18.7 months (95% CI 14.3–23.1) (P = 0.016) in the latter. The median PFS was 13.3 months (95% CI 11.6–14.9) in the LEV group and 12.7 months (95% CI 9.4–16.1) in the VPA group (P = 0.082) (Fig. 1b).

In the multivariate analysis, age (P < 0.001; HR 1.02; 95% CI 1.01–1.03), postoperative KPS score (P = 0.002; HR 0.99; 95% CI 1.01–1.03), complete resection (P < 0.001; HR 0.52; 95% CI 0.69–0.40), methylated *MGMT* promoter (P < 0.001; HR 0.75; 95% CI 0.65–0.87), and LEV use (P = 0.011; HR 0.72; 95% CI 0.55–0.93) were significant prognostic factors for OS (Table 3). Age, postoperative KPS score, complete resection, and methylated *MGMT* promoter were also significant factors influencing the PFS, whereas LEV use was not (P = 0.078; HR 0.80; 95% CI 0.63–1.03).

**Table 3:** Univariate and multivariate analyses of prognostic factors for overall survival and progression-free survival.

	Overall survival				Progression-free survival			
	Univariate		Multivariate		Univariate		Multivariate	
	P value	HR (95% CI)	P value	HR (95% CI)	P value	HR (95% CI)	P value	HR (95% CI)
Age	0.005	1.02 (1.01–1.03)	< 0.001	1.02 (1.01–1.03)	0.007	1.01 (1.00–1.02)	< 0.001	1.02 (1.01–1.03)
Preop. KPS	0.004	0.99 (0.98–1.00)	–	–	0.073	0.99 (0.99–1.00)	–	–
Postop. KPS	< 0.001	0.99 (0.98–0.99)	0.002	0.99 (0.98–1.00)	< 0.001	0.99 (0.98–0.99)	0.001	0.99 (0.98–1.00)
Complete resection	< 0.001	0.55 (0.71–0.43)	< 0.001	0.52 (0.69–0.40)	< 0.001	0.55 (0.44–0.70)	< 0.001	0.49 (0.63–0.37)
Methylated MGMT promoter	0.001	0.78 (0.68–0.91)	< 0.001	0.75 (0.65–0.87)	< 0.001	0.76 (0.67–0.88)	< 0.001	0.72 (0.63–0.83)
LEV use	0.003	0.69 (0.54–0.88)	0.011	0.72 (0.55–0.93)	0.018	0.75 (0.60–0.95)	0.078	0.80 (0.63–1.03)

*HR* hazard ratio, *CI* confidence interval, *KPS* Karnofsky Performance Status, *MGMT* O^6^-methylguanine-DNA methyltransferase, *LEV* levetiracetam.

## Discussion

Although the use of prophylactic AEDs in patients with brain tumour without a seizure is controversial, a survey conducted by the American Association of Neurological Surgeons/Congress of Neurological Surgeons showed that approximately 63% of neurosurgeons used AEDs after brain tumour surgery in patients without a history of seizures^9^. Therefore, some patients are being administered TMZ and an AED together when receiving chemoradiotherapy. Hence, there has been a lot of interest about AEDs that would be beneficial for patients when combined with TMZ.

Phenytoin was the most commonly used AED during the perioperative period until the early 2000s^13, 23^. However, phenytoin induces hepatic cytochrome P450 and has several drug interactions, including interactions with chemotherapy agents^24^. VPA gradually replaced phenytoin, because it has fewer drug interactions^24^. Furthermore, there were reports that VPA extends the survival of patients with GBM^25–30^. The exact mechanism of this survival benefit is unclear; some in vitro studies revealed, as a histone deacetylase, VPA promotes tumour cell differentiation, apoptosis, and growth arrest^31, 32^. Another study showed that VPA down-regulates the expression of MGMT and sensitises glioma cells to TMZ^33^.

Nevertheless, the largest study, which analysed four contemporary randomised clinical trials in newly diagnosed patients with GBM, failed to reveal any survival benefit afforded by the AEDs used^34^. Another large study based on the Cancer Registry of Norway also failed to show survival differences after AED use in 1,263 patients with GBM^35^.

These studies did not provide information about the duration of AED use. According to the AANS/CNS survey, only 16% of neurosurgeons use AEDs for more than 6 weeks^9^. Previous studies may have involved the temporary use of LEV after surgery, which is why LEV may not have affected survival. In our study, the grouping was based on the AEDs administered during the first 2 months, as the time-dependent selection bias could occur on the basis of long-term criteria. As our policy was to maintain the AEDs if there were no significant adverse drug reactions, the AEDs were administered with TMZ during CCRT and the adjuvant chemotherapy period in most patients. The median duration of follow-up and AED use were 60.8 months and 37.3 months, respectively, in our study. Results from the application of the reverse Kaplan–Meier estimator can be interpreted as follows: if a patient had not died, he or she would have been followed for 60.8 months, and AEDs would have been administered for 37.3 months.

The survival analysis showed that the OS and PFS of the LEV (+) group were longer than those of the LEV (−) group. Our findings support the results of the study of Kim et al., in which the survival benefit afforded by LEV in 103 patients with GBM was analysed. Their study was the first clinical study to suggest that LEV affords a survival benefit in patients with GBM. However, the study lacked information about the *IDH* mutation, which can be a confounding factor. We investigated the *IDH1* status in all included patients. To the best of our knowledge, our study is the largest to assess the survival benefit of LEV in patients with *IDH*-wildtype GBMs. Given that *IDH*-wildtype GBMs have poor outcomes compared with *IDH*-mutant GBMs, it is surprising that the Kaplan–Meier curve showed a plateau after 48 months; the 10-year survival rate was estimated to be 22% in the LEV (+) group.

It is not clear how LEV improves the survival of patients with GBM. Bobustuc et al. reported that LEV increases histone deacetylase 1 (HDAC1) transcription and recruits the HDAC1/mSin3A corepressor, which binds to the MGMT promoter region mediated by p53. These three components inhibit MGMT transcription^20^, resulting in TMZ to be more effective. Other in vitro studies have shown LEV enhances the effect of TMZ on GBM stem cell proliferation and apoptosis^36^, and the tumour-inhibition effect of LEV in combination with TMZ in GBM cells^37^.

Recent studies have reported that glioma cells form synapses with neurons and are called as neurogliomal synapses^38, 39^. They revealed that electrical and glutamatergic excitations promote proliferation and invasion of glioma cells, thus making the tumour to progress. From this point of view, it can be hypothesised that the long-term use of AEDs reduces electrical activity and glutamatergic excitation and thus, inhibits the progression of glioma.

The limitation of this study stems from its retrospective nature. As LEV was introduced relatively recently, the LEV (+) group included a more recent cohort of patients. Furthermore, over time, surgeons' skills improved, and the rate of complete resection increased. New technical advances such as the use of 5-aminolevulinic acid also helped to achieve complete resection. Therefore, the LEV (+) group included more patients with complete resection. To overcome this limitation, we conducted a multivariate analysis including well-recognised prognostic factors such as age, KPS score, extent of resection, and MGMT promoter methylation status. The multivariate analysis also showed that the use of LEV had a significant effect on survival in addition to the other prognostic factors.

The seizure frequency after the surgery was not thoroughly investigated in our study. Nevertheless, considering that many patients received monotherapy alone, the seizure rate among our patients would be considerably lower than that reported previously^7, 8^. This can be attributed to the long-term administration of AEDs. LEV was administered for a long time without serious adverse drug reactions.

In this retrospective analysis, the long-term use of LEV was associated with extended OS and PFS rates in patients with GBM. These results suggest that the choice of AED in patients with brain tumours should be carefully considered because it may affect survival. However, as this study had many uncontrolled confounding factors, further prospective controlled studies are needed to prove the association between survival and LEV use in patients with GBM.

## Data Availability

All relevant data are within the paper.

## References

[1] Sanai N, Polley MY, McDermott MW, Parsa AT, Berger MS (2011). An extent of resection threshold for newly diagnosed glioblastomas. J. Neurosurg..

[2] Roh T (2017). Long-term outcomes of concomitant chemoradiotherapy with temozolomide for newly diagnosed glioblastoma patients: A single-center analysis. Medicine.

[3] Stupp R (2005). Radiotherapy plus concomitant and adjuvant temozolomide for glioblastoma. N. Engl. J. Med..

[4] Stupp R (2009). Effects of radiotherapy with concomitant and adjuvant temozolomide versus radiotherapy alone on survival in glioblastoma in a randomised phase III study: 5-year analysis of the EORTC-NCIC trial. Lancet Oncol..

[5] Hegi ME (2005). MGMT gene silencing and benefit from temozolomide in glioblastoma. N. Engl. J. Med..

[6] Esteller M (2000). Inactivation of the DNA-repair gene MGMT and the clinical response of gliomas to alkylating agents. N. Engl. J. Med..

[7] van Breemen MS, Wilms EB, Vecht CJ (2007). Epilepsy in patients with brain tumours: Epidemiology, mechanisms, and management. Lancet Neurol..

[8] van Breemen MSM (2009). Efficacy of anti-epileptic drugs in patients with gliomas and seizures. J. Neurol..

[9] Dewan MC, Thompson RC, Kalkanis SN, Barker FG, Hadjipanayis CG (2017). Prophylactic antiepileptic drug administration following brain tumor resection: Results of a recent AANS/CNS Section on Tumors survey. J. Neurosurg..

[10] Rossetti AO, Stupp R (2010). Epilepsy in brain tumor patients. Curr. Opin. Neurol..

[11] Lwu S, Hamilton MG, Forsyth PA, Cairncross JG, Parney IF (2010). Use of peri-operative anti-epileptic drugs in patients with newly diagnosed high grade malignant glioma: A single center experience. J. Neurooncol..

[12] Patsalos PN (2000). Pharmacokinetic profile of levetiracetam: Toward ideal characteristics. Pharmacol. Ther..

[13] Milligan TA, Hurwitz S, Bromfield EB (2008). Efficacy and tolerability of levetiracetam versus phenytoin after supratentorial neurosurgery. Neurology.

[14] Kern K (2012). Levetiracetam compared to phenytoin for the prevention of postoperative seizures after craniotomy for intracranial tumours in patients without epilepsy. J. Clin. Neurosci..

[15] Fuller KL, Wang YY, Cook MJ, Murphy MA, D'Souza WJ (2013). Tolerability, safety, and side effects of levetiracetam versus phenytoin in intravenous and total prophylactic regimen among craniotomy patients: A prospective randomized study. Epilepsia.

[16] Khan NR (2016). Should levetiracetam or phenytoin be used for posttraumatic seizure prophylaxis? A systematic review of the literature and meta-analysis. Neurosurgery.

[17] Xu JC (2016). The safety and efficacy of levetiracetam versus phenytoin for seizure prophylaxis after traumatic brain injury: A systematic review and meta-analysis. Brain Inj..

[18] Maschio M (2011). Levetiracetam monotherapy in patients with brain tumor-related epilepsy: Seizure control, safety, and quality of life. J. Neurooncol..

[19] Newton HB, Goldlust SA, Pearl D (2006). Retrospective analysis of the efficacy and tolerability of levetiracetam in brain tumor patients. J. Neurooncol..

[20] Bobustuc GC (2010). Levetiracetam enhances p53-mediated MGMT inhibition and sensitizes glioblastoma cells to temozolomide. Neuro. Oncol..

[21] Kim Y-HH (2015). Survival benefit of levetiracetam in patients treated with concomitant chemoradiotherapy and adjuvant chemotherapy with temozolomide for glioblastoma multiforme. Cancer.

[22] Wen PY (2010). Updated response assessment criteria for high-grade gliomas: Response assessment in neuro-oncology working group. J. Clin. Oncol..

[23] Chang EF (2008). Seizure characteristics and control following resection in 332 patients with low-grade gliomas. J. Neurosurg..

[24] Vecht CJ, Wagner GL, Wilms EB (2003). Interactions between antiepileptic and chemotherapeutic drugs. Lancet Neurol..

[25] Oberndorfer S (2005). P450 enzyme inducing and non-enzyme inducing antiepileptics in glioblastoma patients treated with standard chemotherapy. J. Neurooncol..

[26] Weller M (2011). Prolonged survival with valproic acid use in the EORTC/NCIC temozolomide trial for glioblastoma. Neurology.

[27] Kerkhof M (2013). Effect of valproic acid on seizure control and on survival in patients with glioblastoma multiforme. Neurol. Oncol..

[28] Yuan Y (2014). Survival analysis for valproic acid use in adult glioblastoma multiforme: A meta-analysis of individual patient data and a systematic review. Seizure.

[29] Redjal N (2016). Valproic acid, compared to other antiepileptic drugs, is associated with improved overall and progression-free survival in glioblastoma but worse outcome in grade II/III gliomas treated with temozolomide. J. Neurooncol..

[30] Watanabe S (2017). Valproic acid reduces hair loss and improves survival in patients receiving temozolomide-based radiation therapy for high-grade glioma. Eur. J. Clin. Pharmacol..

[31] Shao Y, Gao Z, Marks PA, Jiang X (2004). Apoptotic and autophagic cell death induced by histone deacetylase inhibitors. Proc. Natl. Acad. Sci. USA.

[32] Knupfer MM (2001). Different effects of valproic acid on proliferation and migration of malignant glioma cells in vitro. Anticancer Res..

[33] Ryu C (2012). Valproic acid downregulates the expression of MGMT and sensitizes temozolomide-resistant glioma cells. J. Biomed. Biotechnol..

[34] Happold C (2016). Does valproic acid or levetiracetam improve survival in glioblastoma? A pooled analysis of prospective clinical trials in newly diagnosed glioblastoma. J. Clin. Oncol..

[35] Knudsen-Baas KM, Engeland A, Gilhus NE, Storstein AM, Owe JF (2016). Does the choice of antiepileptic drug affect survival in glioblastoma patients?. J. Neurooncol..

[36] Scicchitano BM (2018). Levetiracetam enhances the temozolomide effect on glioblastoma stem cell proliferation and apoptosis. Cancer Cell Int..

[37] Marutani A (2017). Tumor-inhibition effect of levetiracetam in combination with temozolomide in glioblastoma cells. Neurochem. J..

[38] Venkataramani V (2019). Glutamatergic synaptic input to glioma cells drives brain tumour progression. Nature.

[39] Venkatesh HS (2019). Electrical and synaptic integration of glioma into neural circuits. Nature.

